# Zinc Vanadate (Zn_3_V_2_O_8_) Immobilized Multiwall Carbon Nanotube (MWCNT) Heterojunction as an Efficient Photocatalyst for Visible Light Driven Hydrogen Production

**DOI:** 10.3390/molecules28031362

**Published:** 2023-01-31

**Authors:** Fahad A. Alharthi, Alanood Sulaiman Ababtain, Hamdah S. Alanazi, Alanoud Abdullah Alshayiqi, Imran Hasan

**Affiliations:** Department of Chemistry, College of Science, King Saud University, Riyadh 11451, Saudi Arabia

**Keywords:** MWCNT, Nanocomposites, H_2_ evolution, heterojunction, photocatalytic process

## Abstract

Z-scheme photocatalytic reaction is considered an effective strategy to promote the photogenerated electron-hole separation for significantly improving the efficiency of photocatalytic hydrogen precipitation from splitting water. In this study, a heterojunction nanocomposite material based on Zn_3_V_2_O_8_ (ZV) with MWCNT was prepared by a hydrothermal process. The photocatalysts were characterized by X-ray diffraction, scanning electron microscopy (SEM), Fourier transform infrared (FTIR), UV-visible absorption spectroscopy, and transmission electron microscopy (TEM) to understand crystal structure, morphology, and optical properties. The efficiency of the samples was evaluated for the photocatalytic H_2_ production under visible solar radiation using water glycerol as a sacrificial reagent. The obtained results suggest that, between ZV and ZV@MWCNT, the latter shows higher efficiency for H_2_ production. The maximum H_2_ production efficiency was found to be 26.87 μmol g^−1^ h^−1^ for ZV and 99.55 μmol g^−1^ h^−1^ for ZV@MWCNT. The synergistic effect of MWCNT to ZV resulted in improving the efficiency of charges and light-absorbing capacity, resulting in enhanced H_2_ production in the heterojunction nanocomposite material. The nanocomposite was stable and highly efficient for H_2_ production of six or more cycles. Based on the outcomes of this study, it can be observed that forming the heterojunction of individual nano systems could result in more efficient material for H_2_ production under visible solar energy.

## 1. Introduction

Environmental issues and global energy demands have increasingly evolved as major world concerns. To address these issues, economic and eco-friendly sources of renewable and clean energy have become a matter of research in recent years [1,2]. Various methods produce hydrogen, such as electrolyzed water, biomass gasification, fermentation, and steam reforming, but high electricity consumption limits their efficiency [3,4]. Hydrogen production through photocatalytic water splitting has therefore been recognized as one promising and green method owing to its highly efficient utilization of solar energy [5,6]. The hydrogen energy produced by this method has been regarded as the most sustainable energy due to its high calorific values and carbon-free combustion products [7]. Since the efficiency of this method greatly depends upon the effective use and maximum absorption of solar energy, there is a need to develop a semiconductor photocatalyst with regulated electronic structure, lattice structure, and photocatalytic activity [8,9].

The literature shows that various semiconductor materials, such as CdS [10], TiO_2_ [11], MoS_2_ [12], BiVO_4_–ZnCdS [13], ZnIn_2_S_4_ [14], and oxysulfides (OS) including MoCuOS [15], Zn-NiOS [16], Al_2_OS [17], and Zn-Ce-GaOS [18], have been developed for photocatalytic hydrogen production. Among these materials, metal vanadates with general formulae (M_3_V_2_O_8_; M = Zn, Cd, Ni, Cu, Co, etc.) have gained considerable attention due to their potential applications in implantable materials, catalysis, low-temperature magnetic devices, and batteries [19,20]. Zinc vanadate (Zn_3_V_2_O_8_; ZV) is one of the most versatile and important members of the vanadate family, having extensive uses in various applications such as optical devices, sensors, catalysts, and cathodes [21,22]. Recently, vanadates have been reported as the most efficient materials for photocatalytic hydrogen production [23,24,25]. The electronic structure of these vanadates, especially in the case of Cu and Fe, consists of O 2p orbitals as valence bands (VB) and partially filled 3d orbitals as conduction bands (CB) which improve the light absorption and thus the water splitting process [25,26]. However, in the case of ZV, the VB is O 2p orbital, but the CB is 3d orbital from Vanadium because of the filled 3d orbital of zinc, resulting in a high rate of electron-hole recombination inhibiting its photocatalytic activity [27].

Carbon nanotubes (CNTs) are considered good supporting materials for various semiconductor photocatalysts to boost their photocatalytic activity through their unique electronic properties, large surface areas, and large electron storage capacities [28,29]. As compared to SWCNTs (single wall) or DWCNTs (double wall), MWCNTs have been found to be more efficient in hydrogen production due to enhanced electrical conductivity, high aspect ratios, and optical properties [30]. The present study focuses on the hydrothermal synthesis of a heterojunction Zn_3_V_2_O_8_/MWCNT material for enhanced photocatalytic water splitting and hydrogen production applications using 10% aqueous glycerol as a sacrificial agent under a visible light source. The material was characterized for its morphological, optical, structural, and elemental analysis to evaluate the synergistic effect of MWCNT on ZV for H_2_ production.

## 2. Results and Discussion

### 2.1. Material Characterization Studies

**Figure 1:**
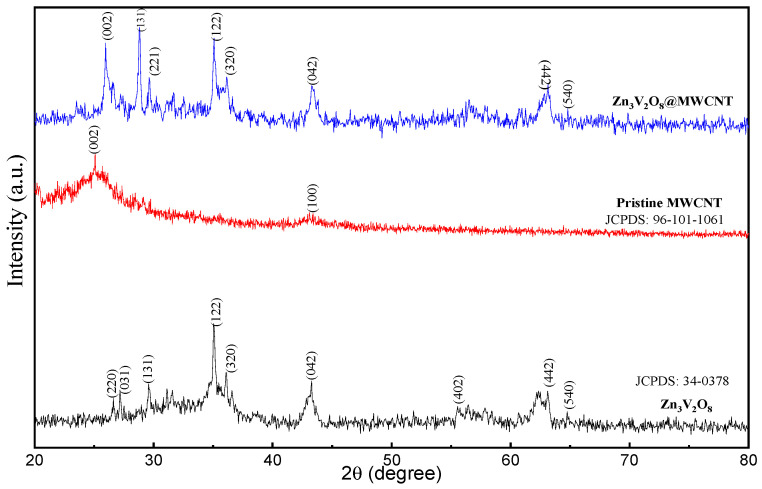
XRD spectra of ZV (black line) and ZV@MWCNT (red line) calcined at 550 °C.

Figure 1 represents the XRD spectra of ZV and ZV@MWCNT calcined at 550 °C in the range of 10–80° (2θ angle). The XRD spectra of ZV exhibits characteristic peaks at 2θ values of 26.49, 27.20, 29.59, 35.07, 36.20, 43.23, 55.59, 63.26, and 64.86 which correspond to Miller indices values of (220), (031), (131), (122), (320), (042), (402), (442), and (540) simulated with JCPDS card no. 34–0378. The XRD data suggested the nanomaterials associated with an orthorhombic phase structure. The XRD spectra of pristine MWCNT exhibit two characteristic hkl planes at (002) and (100) JCPDS No. 96-101-1061. The XRD spectra of ZV@MWCNT also exhibited maximum peaks from ZV except for the peak at 26.00 belonging to Miller indices values (002) associated with MWCNT [31]. The formation of the heterojunction of ZV with MWCNT shifted the intense peak from 35.07 to 28.82 degrees, resulting in the increase in interplanar distance 2.56 Å to 3.09 Å. Using the Scherer equation, the crystallite size was calculated as 32.65 nm for ZV and 22.83 nm for ZV@MWCNT. The contraction in size after heterojunction formation resulted in a reduction in the bandgap and thus enhanced photocatalytic activity will be delivered by the synthesized nanomaterial. The crystallinity of the material also increased from 45.60% to 50.90%. The XRD results clearly suggested that ZV was successfully functionalized by MWCNT.

**Figure 2:**
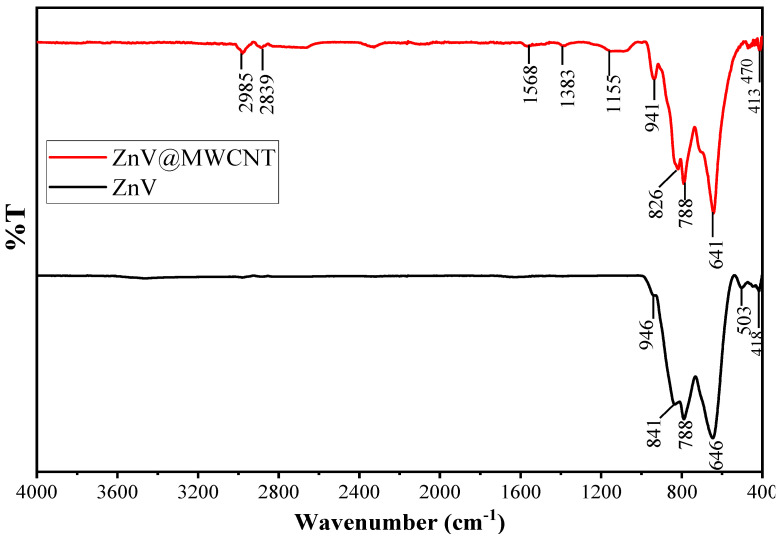
FTIR spectra of ZV (black line) and ZV@MWCNT (red line) calcined at 550 °C.

The FTIR spectrum of ZV and ZV@MWCNT has been given in Figure 2. The FTIR spectra of ZV exhibit characteristic peaks at 946, 841, and 646 cm^−1^, belonging to metal group V-O-V, stretching vibrations in a tetrahedral rocking vibration mode of VO_4_, 788 cm^–1,^ due to bonds shared by corner atoms of VO_4_ tetrahedral structure, 503 and 418 cm^−1^, resulting from stretching vibrations of V–O–Zn [21]. The FTIR spectra of ZV@MWCNT exhibited most of the peaks from ZV with some shifted wave number values, except for 2985, 2839 cm^−1^ belonging to stretching vibrations of -CH_2_ groups, 1568 cm^−1^ due to the aromatic C=C type bonds, 1383, and 1155 cm^−1^ CH_2_ bending and wagging vibrations in graphite structure of MWCNT [28]. The presence of these peaks confirms that ZV has been successfully functionalized by MWCNT to form a heterojunction ZV@MWCNT. Since the synthesized compounds are calcined as 550 °C, there is no peak of absorbed water (–OH).

**Figure 3:**
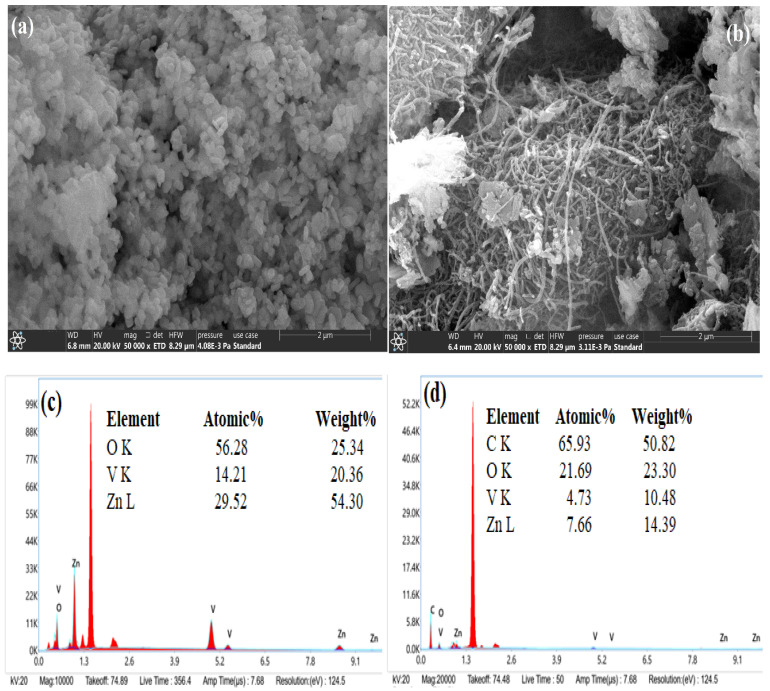
SEM image of (**a**) ZV, (**b**) ZV@MWCNT, (**c**) EDX spectra of ZV, and (**d**) EDX spectra of ZV@MWCNT.

**Figure 4:**
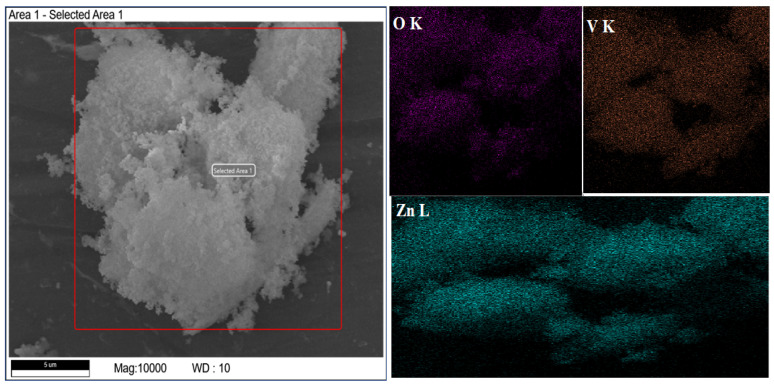
Elemental mapping of ZV.

**Figure 5:**
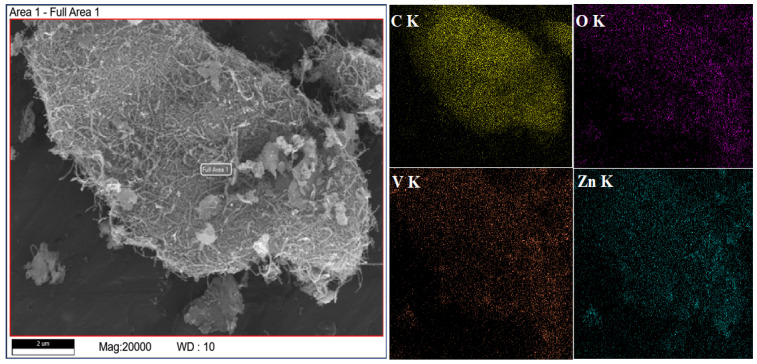
Elemental mapping of ZV@MWCNT.

The surface morphology, approximate particle size, and elemental mapping of the as-synthesized material were accomplished using SEM–EDX analysis. The results obtained are given in Figure 3a–d. The SEM image of ZV (Figure 3a) represents an assembly of intertwined platelets growing in all directions with particle sizes in the range of nm while the SEM image of ZV@MWCNT (Figure 3b) exhibits a wire-like morphology belonging to MWCNT associated with flakes. The EDX spectra given in Figure 3c,d confirm the peaks of various elements in ZV and ZV@MWCNT. The elemental mapping given in Figure 4 and Figure 5 confirms the uniform distribution of all of the elements in the materials with atomic (%) as O (56.28), Zn (29.52), and V (14.21) in ZV, and with C (65.93), O (21.69), Zn (7.66), and V (4.73) in ZV@MWCNT. No other impurity appears in the sample which is supported by elemental mapping. The results supported the formation of the heterojunction between ZV and MWCNT.

**Figure 6:**
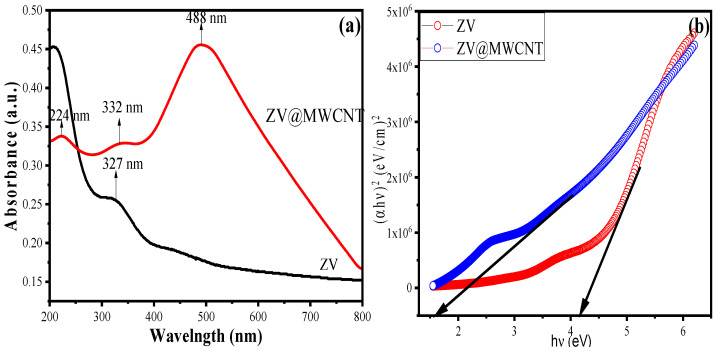
(**a**) UV-Vis spectra of ZV and ZV@MWCNT (**b**) corresponding Tauc’s plot for calculation of energy bandgap.

UV–Vis spectroscopy was employed to observe the optical properties of the synthesized material. The results obtained are given in Figure 6a,b. The UV spectra of ZV in Figure 6a exhibited a single peak at 327 nm, suggesting that the material is UV light-active, and the absorption band belongs to the charge transfer from Zn–O–V bonds. The UV spectra of ZV@MWCNT (Figure 6a red line) exhibits three absorption bands at 224 nm, 332 nm, and 488 nm. This result suggests that the synthesized material is both UV and visible light-active. The formation of the heterojunction of ZV with MWCNT has enhanced the light-absorbing properties of the material, and the appearance of broadband in UV spectra indicates the size of the particles in the nanometer range. The absorption peak at 224 nm belongs to the absorption energy of π electrons in the MWCNT structure, 332 nm belongs to Zn–O charge transfer, and 488 nm belongs to V–O charge transfer in the tetrahedral VO_4_^3−^ structure [32,33]. More information about the bandgap energy was elaborated using Tauc’s plot, given mathematically as

(1) $$(\alpha h\nu )=A(h\nu -E_{g}{)}^{n}$$

where h = plank constant, α= absorption coefficient, A = constant, and ν = frequency of radiations with n being the constant of transition variation corresponding to n = 2 in direct transitions and n = 1/2 in indirect transitions. Using Equation (1), Tauc’s plot has been given in Figure 6b in which the energy bandgap for ZV was found to be 4.15 eV while ZV@MWCNT was 1.59 eV. Contraction in the energy bandgap after heterojunction formation, therefore, was reflected in the enhanced optical properties.

**Figure 7:**
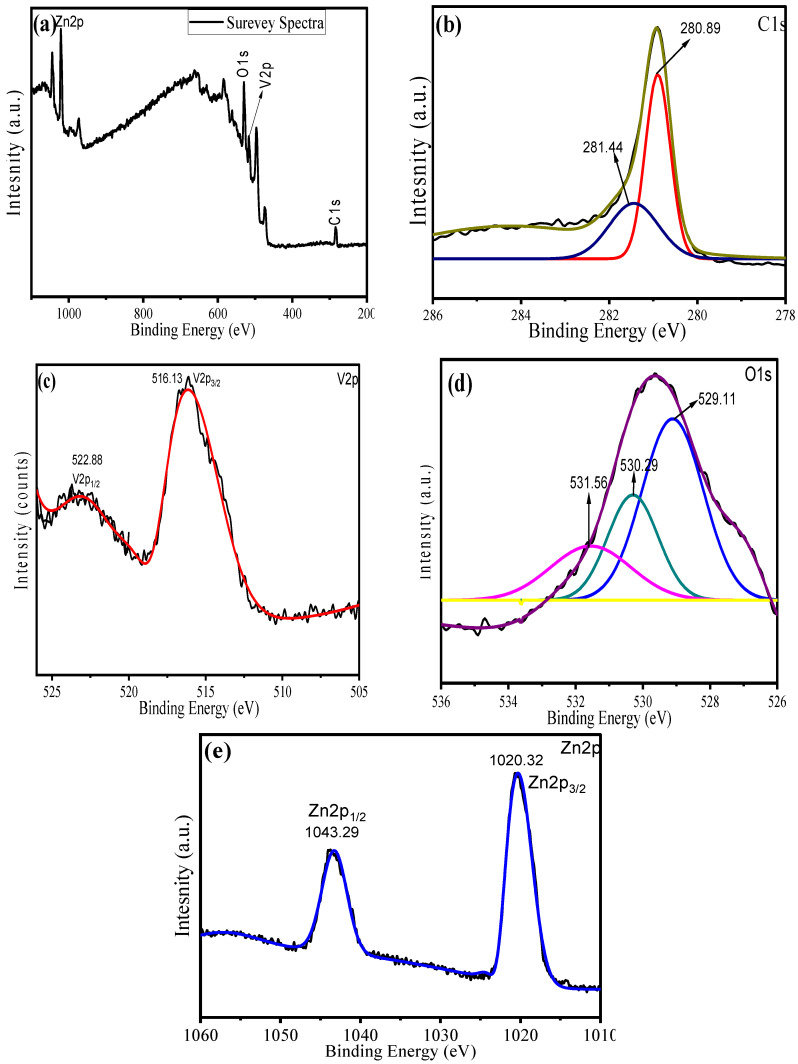
(**a**) XPS survey spectra of ZV@MWCNT (**b**) C1s (**c**) V2p (**d**) O1s and (**e**) Zn2p.

Further XPS analysis was performed to observe the surface electronic and chemical states of the elements along with binding energy. The results are given in Figure 7a–e. Figure 7a represents the survey spectra of ZV@MWCNT which confirm the characteristic peaks at 284.9 eV, 515 eV, 528.11 eV, and 1020 eV ascribed to the presence of C1s, V2p, O1s, and Zn2p. The atomic concentration table confirms the elemental composition as C (51.25%), O (30.82%), V (6.55), and O (11.37%). Figure 7b represents the deconvoluted XPS spectra of C1s with peaks at 280.89 and 281.44 eV belonging to C=C, C–O, and CH_2_ aromatic (graphitic carbon) in the MWCNT matrix [34,35]. These results confirm, therefore, that MWCNT is attached to the surface of ZV through the formation of C–O bonds. The deconvoluted XPS spectra of V2p are given in Figure 3c which exhibits two peaks at 516.13 eV and 522.88 eV belonging to the V2p_3/2_ and V2p_1/2_ states with spin-orbit splitting of 6.75 eV associated with the V^5+^ state [36]. The XPS spectra of O1s in Figure 7d show deconvoluted peaks at 529.11, 530.29, and 531.51 eV associated with the V–O bond, Zn–O bond, and C–O bond, along with a large number of defect sites with minimum oxygen coordination and small particle size [37]. The XPS spectra of Zn2p exhibit two deconvoluted peaks at 1020.32 eV and 1043.29 belonging to Zn2p3/2 and Zn2p1/2 state Zn^2+^ ions [38].

**Figure 8:**
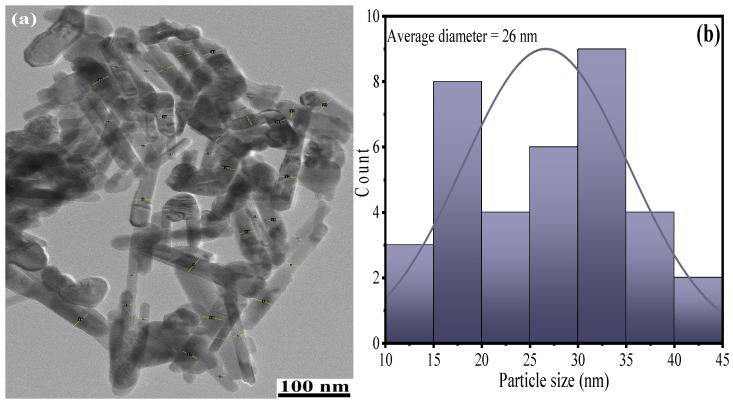
(**a**) Transmission electron microscopic (TEM) image of ZV@MWCNT and (**b**) gaussian distribution curve for average particle distribution.

Further, the shape of the particles, their orientation, and their distribution in the material matrix were evaluated by transmission electron microscopy (TEM) analysis. Figure 8a represents the TEM image of ZV@MWCNT which shows the randomly oriented nanorods distributed in the MWCNT matrix. The average diameter of the nanorods was calculated as 26 nm using the gaussian distribution graph given in Figure 8b which is found to be in close agreement with Scherer’s calculated crystallite size.

### 2.2. Hydrogen Production Experiments

#### 2.2.1. Selection of Sacrificial Reagent

**Figure 9:**
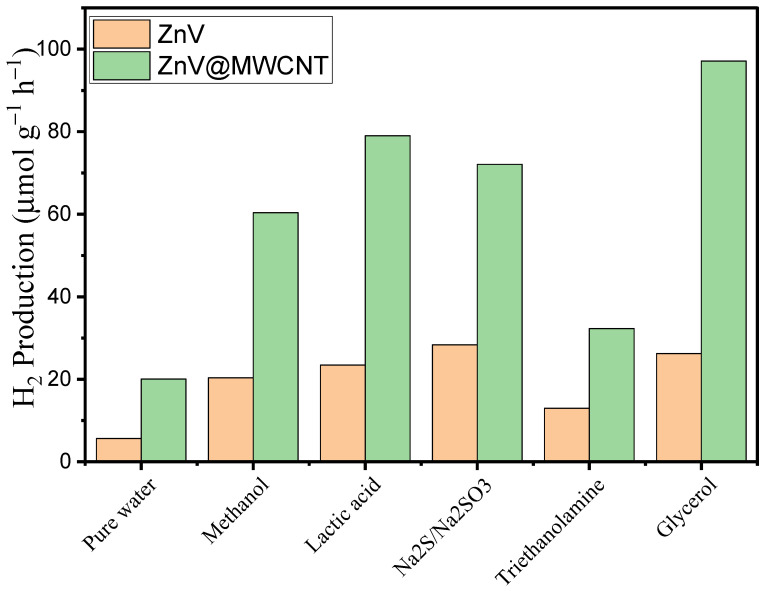
Effects of various sacrificial reagents on ZV and ZV@MWCNT for PC H_2_ production reaction.

The sacrificial reagent (SR) in the photocatalytic hydrogen (PC H_2_) production reaction plays the most vital role in the water-splitting process. Various SRs such as pure water, methanol, lactic acid, Na_2_S/Na_2_SO_3_, triethylamine, and glycerol were taken with ZV and ZV@MWCNT in a photochemical reactor under a visible light source. The results obtained are given in Figure 9 which shows that with pure water the rate of H_2_ production over ZV was 5.63 μmol g^−1^ h^−1^, for methanol 20.35 μmol g^−1^ h^−1^, for lactic acid 23.42 μmol g^−1^ h^−1^, for Na_2_S/Na_2_SO_3_ 28.35 μmol g^−1^ h^−1^, for triethylamine 13.21 μmol g^−1^ h^−1^, and for glycerol 27.75 μmol g^−1^ h^−1^. The ZV@MWCNT rate of H_2_ production was found to be 20.06 μmol g^−1^ h^−1^, for methanol 60.38 μmol g^−1^ h^−1^, for lactic acid 79.03 μmol g^−1^ h^−1^, for Na_2_S/Na_2_SO_3_ 72.10 μmol g^−1^ h^−1^, for triethylamine 32.31 μmol g^−1^ h^−1^, and for glycerol 97.13 μmol g^−1^ h^−1^. The results showed that glycerol produced the maximum amount of H_2_ for both ZV and ZV@MWCNT. Glycerol was chosen, therefore, as the sacrificial reagent for all the PC H_2_ production experiments. The results also suggested that the formation of heterojunction ZV with MWCNT resulted in enhanced PC H_2_ production efficiency by rendering enhanced photocarrier generation and increased active sites. Glycerol is a byproduct of biodiesel production that can be easily photo-reformed in an aqueous solution at ambient temperatures as compared to other sacrificial reagents [39]. Moreover, 1 mole of glycerol can result in 7 moles of H_2_ gas given by the global reaction (Equation (2)) [40]:

(2) $$C_{3}H_{8}O_{3}+H_{2}O\to 7H_{2}\left(g\right)+3CO_{2}(g)$$

This reaction is quite environmentally friendly and can happen at room temperature in a single step to produce H_2_ and CO_2_ gas with low levels of CO gas.

#### 2.2.2. Effects of Various Reaction Parameters

**Figure 10:**
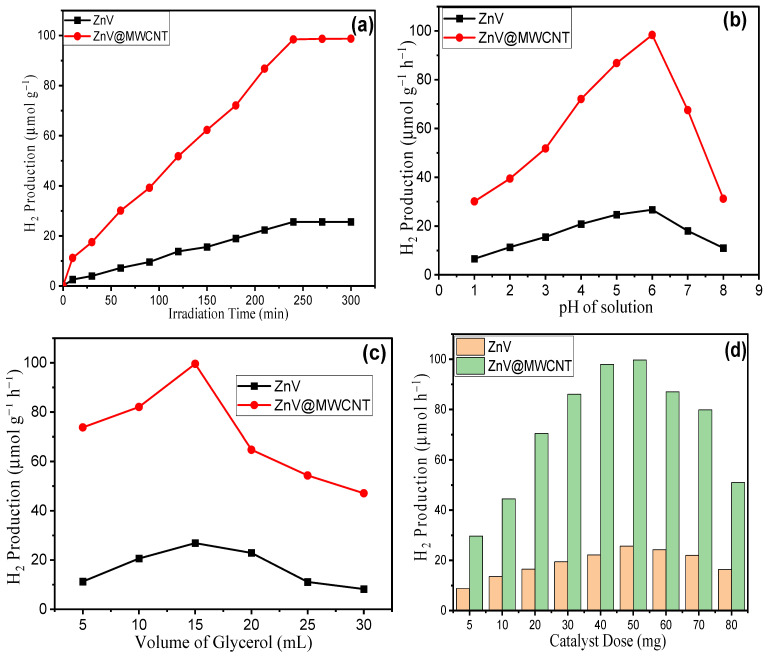
Effects, observation, and optimization of (**a**) irradiation time, (**b**) pH of the media, (**c**) concentration of glycerol, and (**d**) catalyst dose.

The effects of various parameters such as irradiation time, pH of the media, concentration of glycerol (mL), and catalyst dose (mg) were observed on the rate of PC H_2_ production under a visible light source. In this regard, PC experiments were performed by taking 10% glycerol in water with 50 mg of synthesized nanomaterials (ZV, ZV@MWCNT) for a time interval from 0 to 5 h. The results given in Figure 10a suggest that, as the irradiation time increases from 0 to 5 h, the rate of H_2_ production also increases until 4 h (240 min) for both the catalyst materials. After that, the rate becomes constant. The trend can be explained on the basis that the increase in irradiation time results in a high rate of photo-absorption, then in generating a greater number of ^•^OH or ^•^O_2_^−^ radicals responsible for the water splitting process [41]. The attainment of a constant rate beyond 4 h reflects on the accumulation of all the surface-active sites of the catalyst. The rate of H_2_ production for ZV was found to be 25.60 μmol g^−1^ h^−1^, while for ZV@MWCNT it was 98.75 μmol g^−1^ h^−1^. The higher rate of H_2_ production by ZV@MWCNT results from its enhanced optical properties through heterojunction formation.

Next, the effect of the pH of media on the rate of PC H_2_ production was observed for both ZV and ZV@MWCNT. Fifty mg of the synthesized catalyst were dispersed in 100 mL of 10% glycerol water solution with variable pH from one to eight under visible light irradiation of 5 h. The results obtained are given in Figure 10b. The results indicate that as the pH increases from one to six, an increase in PC H_2_ production results in a maximum value of 26.67 μmol g^−1^ h^−1^ for ZV and 98.35 μmol g^−1^ h^−1^ for ZV@MWCNT. With an increase in pH from one to six, there is an increase in the rate of H_2_ production which then declines as the pH increases beyond six. At a lower pH value, a higher number of H^+^ ions will be deviated by the positive surface of the catalyst and thus result in a low H_2_ production rate. As the pH increases, however, the number of H^+^ decreases, resulting in a slow rate of H_2_ production [42]. With the heterojunction ternary nanocomposite structure formed, the separation and transfer of photocarriers are facilitated, their lifetime in the PC system is extended, and the recombination process is retarded, thus increasing the H_2_ production efficiency of the ternary nanocomposite.

The variation in concentration of sacrificial reagent glycerol (Vol) was examined on both ZV and ZV@MWCNT. These results are depicted in Figure 10c. It can be seen that the rate of PC H_2_ production was 11.25 μmol g^−1^ h^−1^ at 5 mL, 20.63 μmol g^−1^ h^−1^ at 10 mL, 28.88 μmol g^−1^ h^−1^ at 15 mL, 22.88 μmol g^−1^ h^−1^ at 20 mL, 11.10 μmol g^−1^ h^−1^ at 25 mL, and 8.23 μmol g^−1^ h^−1^ at 30 mL of glycerol volume over ZV. Similarly, over ZV@MWCNT, the rate of PC H_2_ production was found to be 73.75 μmol g^−1^ h^−1^ for 5 mL, 82.05 μmol g^−1^ h^−1^ for 10 mL, 99.55 μmol g^−1^ h^−1^ for 15 mL, 64.75 μmol g^−1^ h^−1^ for 20 mL, 54.32 μmol g^−1^ h^−1^ for 25 mL, and 47.12 μmol g^−1^ h^−1^ for 30 mL of glycerol. Therefore, 15 mL of glycerol was chosen as the optimum volume of sacrificial reagent to produce a maximum rate of H_2_ production.

Finally, the effect of the catalyst dose was observed by taking 15 mL of glycerol with 85 mL of water at pH 6 with a variable number of catalysts, such as 10 to 100 mg under visible light irradiation of 4 h. The results obtained after the completion of the PC reaction are given in Figure 10d. The results show that, with an increase in catalyst dose from 10–50 mg, the rate of H_2_ production also increases, attaining a maximum value of 25.65 μmol h^−1^ for ZV and 99.69 μmol h^−1^ for ZV@MWCNT. A further increase in the catalyst dose beyond 50 mg results in a decrease in the rate of the reaction. The trend can be explained by the fact that, as the catalyst dose increases, a greater number of active sites can absorb the radiation and generate photogenerated charge carriers for the water splitting process. However, beyond a limit, the solution turns turbid due to the agglomeration of nanoparticles which scatter the photon radiation, reaching the surface of the catalyst and thus hindering the rate of H_2_ production [43].

### 2.3. Reusability Experiments

**Figure 11:**
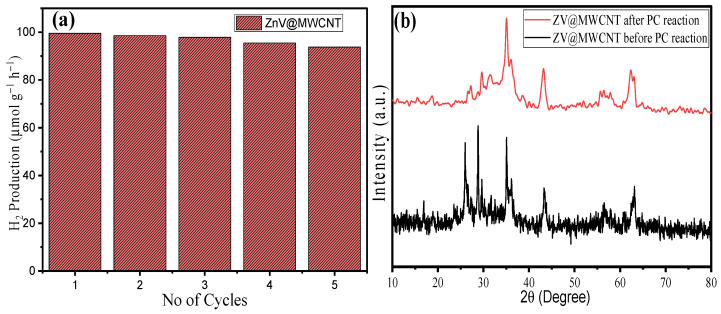
(**a**) Reusability plot for ZV@MWCNT towards PC H_2_ production and (**b**) corresponding XRD spectra of the material before and after five cycles of application.

The performance of a photocatalyst greatly depends upon its stability and reusability for a particular application. Therefore, experiments were performed for the usability of the ZV@MWCNT towards PC H_2_ production. The results obtained are given in Figure 11a. After the reaction was completed, the material was taken out of the PC system, collected through the centrifuge, washed with deionized water, and dried in a hot air oven. Similarly, the same procedure was applied for the second through the fifth cycles. The results show that the material ZV@MWCNT is highly stable as no considerable change in H_2_ production efficiency occurs over five cycles of use. The change in crystal structure or purity of the material was investigated by XRD analysis, the results of which are given in Figure 11b. The XRD spectra show the same diffraction peaks as those exhibited by pure synthesized material but with reduced intensity. These results suggest that the synthesized material is highly stable and reusable after five cycles for PC H_2_ production applications using glycerol as a sacrificial agent.

### 2.4. Mechanism of H_2_ Production

**Scheme 1:**
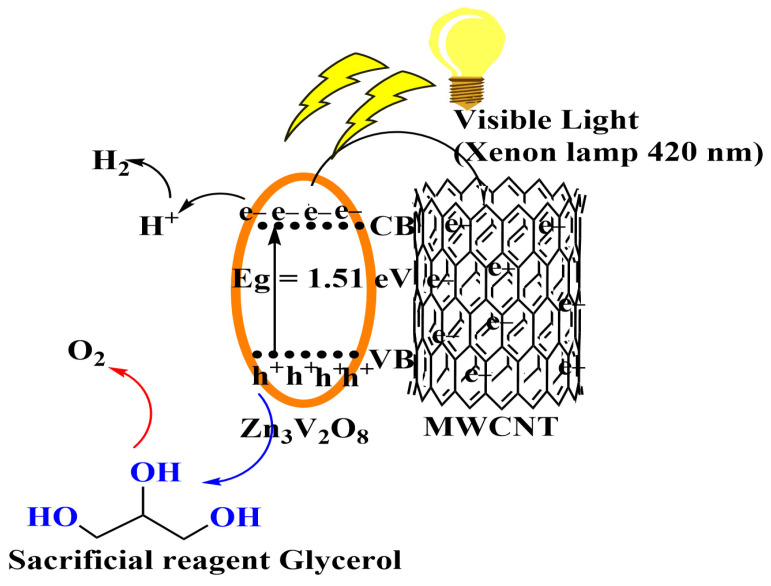
Illustration of water-splitting and glycerol oxidation/reforming reactions over the ZV@MWCNT photocatalyst under light irradiation.

Scheme 1 represents the plausible mechanism for the water splitting and a glycerol oxidation/reforming reaction over ZV@MWCNT NC under light irradiation. From the optical studies, it was found that the incorporation of MWCNT with ZV results in a decrease in the energy bandgap and an increase in light-absorption properties. This finding supports the experimental result that ZV@MWCNT has higher activity than pristine ZV. The irradiation of the material resulted in the formation of photogenerated electron-hole pairs (Equation (3)). The photogenerated electron can move from CB of ZV to MWCNT, and, being an e-trapper, MMCNT can trap the e- and enhance the separation of photogenerated electrons and holes [44]. The holes formed in the valence band (VB) react with water to generate hydroxyl radicals (^•^OH) and H^+^ ions (Equation (4)). The H^+^ ions react with photogenerated e- on the surface of MWCNT and thus decompose into H_2_ gas (Equation (5)). The hydroxyl radicals react with glycerol to produce H_2_O and CO_2_ (Equation (6)). Thus, glycerol can suppress the electron-hole pair recombination rate by reacting with hydroxyl radicals and stabilizing the photogenerated holes, hence increasing hydrogen production efficiency [45]. It can be seen that both photocatalytic water splitting and glycerol photo reforming contribute towards H_2_ gas production. The formation of heterojunction ZV@MWCNT, therefore, results in higher H_2_ production rate from the glycerol-water system [46].

(3) $$\mathrm{ZV}@\mathrm{MWCNT}\overset{hv}{\to }\mathrm{ZV}@\mathrm{MWCNT}*{(h}_{\mathrm{VB}}^{+}+e_{CB}^{-})$$

(4) ZV@MWCNT*(hVB+)+H2O→H++O•H

(5) $$\mathrm{ZV}@\mathrm{MWCNT}*{(e}_{CB}^{-}{)+H}^{+}\to {1/2H}_{2}$$

(6) O•H+Glycerol→H2O+CO2

## 3. Materials and Methods

### 3.1. Reagents and Chemicals

Zinc nitrate (Zn (NO_3_)_2_.4H_2_O, 99%), Vanadium pentoxide (V_2_O_5_), and Multiwall carbon nanotubes (MWCNT, >98%) were purchased from Merck Company. Hydrogen peroxide (H_2_O_2_), sodium hydroxide (NaOH Pellets), and ethanol were supplied by Loba Chemie. All of the chemicals were used as received without further purifications.

### 3.2. Synthesis of ZV and ZV@MWCNT

The pristine ZV and ZV@MWCNT were synthesized by the hydrothermal method with some modifications [47]. In a 100 mL capacity beaker, 0.50 g V_2_O_5_ dissolved in 25 mL of DI water was added followed by the addition of 30% H_2_O_2,_ drop by drop. The color of the solution changed to orange from bright yellow (Sol A). In another vessel, an aliquot of 0.025 g MWCNT dispersed in 20 mL of DI water was taken and sonicated for 30 min at 25 °C (Sus B). Suspension B was added to solution A, and the mixture was left on a magnetic stirrer for 2 h. After 2 h, an amount of 0.15 g of Zn (NO_3_)_2_.6H_2_O in 10 mL of DI water was added to the above mixture and continued to mix under magnetic stirring for another 30 min. After 30 min, 0.2 M solution of NaOH was added to the above mixture to maintain the pH 8–9. This mixture was transferred to a 100 mL Teflon-lined autoclave and heated at 190 °C for 18 h. After the completion of the reaction, the autoclave was allowed to cool naturally at room temperature, and the precipitate was obtained by centrifugation. The material was washed several times using DI water and ethanol to remove any unreacted reactant species, dried at 90 °C for 4 h in a hot air oven, and finally calcined at 550 °C for 4 h with a heating rate of 5 °C/min. Similarly, pristine ZV was also prepared by the same method without using MWCNT.

### 3.3. Instrumentation Used

The synthesized samples ZV and ZV@MWCNT were made to undergo thorough morphological, structural, elemental, optical, and chemical tests to evaluate the properties of the synthesized material ZV@MWCNT as compared to pristine ZV. For example, the structural analysis was done by using a Rigaku Ultima 1 V X-ray diffractometer (XRD, Austin, TX, USA) and Perkin Elmer Spectrum 2 FTIR spectrometer (Massachusetts, USA). The morphological studies were carried out by SEM (JEOL GSM 6510LV, Tokyo, Japan) and TEM (JEM-2100, Tokyo, Japan). The optical studies were carried out by a Shimadzu UV-1900 double-beam UV-Vis spectrophotometer (Kyoto, Japan). X-ray photoelectron spectroscopy (XPS, PHI 5000 Versa Probe III, Physical Electronics, Chanhassen, MN, USA.) was applied to check the chemical state of the individual elements in the material.

### 3.4. Photocatalytic Hydrogen Production Experiments

A comparative study was carried out between ZV and ZV@MWCNT to evaluate the photocatalytic H_2_ production efficiency in a solution of water glycerol as a sacrificial reagent. The reaction was carried out in a photocatalytic reactor equipped with a double-walled quartz reaction vessel connected to a closed gas circuit with a water jack flushing cold water to maintain the temperature of the reaction at 10 °C. Before the start of the reaction, the reaction mixture was bubbled through argon gas to remove oxygen from it and maintain an anaerobic condition. After the bubbling process, photocatalytic experiments were carried out by taking 50 mg of synthesized material ZV and ZV@MWCNT dispersed in 80 mL of water + 20 mL of glycerol under a visible light source (Xe lamp 420 nm). The produced H_2_ gas was analyzed by a multichannel analyzer (Emerson) equipped with a thermal conductivity detector.

## 4. Conclusions

In the present study, a nanocomposite heterojunction based on zinc vanadate multiwalled carbon nanotube (ZV@MWCNT) was synthesized by hydrothermal reaction. The structural studies suggested an orthorhombic phase structure associated with 22.83 nm of crystallite size and 3.09 A of interplanar distance at a (131) Miller index value for the synthesized nanomaterial. The XPS studies suggested the formation of heterojunction between ZV and MWCNT through C–O linkage. The data from EDX and XPS analysis are found in good agreement with the composition and chemical state of the elements constituting the nanomaterial based on binding energy values. The UV-Vis spectroscopy suggested the material to be visible light-active with an energy bandgap value of 1.59 eV. The synthesized nanomaterials were applied as photocatalysts for H_2_ production through a water-splitting process using 15% glycerol/water composition as a sacrificial reagent under a visible light source. The rate of H_2_ production for pristine ZV and ZV@MWCNT was found to be 26.87 μmol g^−1^ h^−1^ and 99.55 μmol g^−1^ h^−1^ respectively at pH 6 using 50 mg of catalyst for 4 h of visible light irradiation. The material ZV@MWCNT was found to be highly stable and reusable, even after five cycles of use. The outcomes of this study suggested that the formation of heterojunctions between ZV and MWCNT resulted in the inhibition of the fast recombine of electron-hole pairs, thus increasing the lifetime for light absorption which is reflected in the high rate of H_2_ production by the material.

## Data Availability

Data is contained within the article.

## Sample Availability

Samples of the compounds Zinc vanadate and Zinc vanadate@MWCNT are available from the authors.
